# Association Between Smartphone-Based IMU Turning Performance and Screening-Defined Mild Cognitive Impairment in Older Adults

**DOI:** 10.3390/s26185795

**Published:** 2026-09-12

**Authors:** Masayuki Hoshi, Koei Ishida, Ayane Nakamaru, Yuu Okabe, Misuzu Kusano, Yui Miyazaki, Tatsuya Nakanowatari, Toshimi Sato, Akihiko Asao, Natsumi Kimura, Maki Ogasawara, Hiroshi Hayashi, Toshimasa Sone, Yoshitaka Shiba

**Affiliations:** 1 Department of Physical Therapy, School of Health Sciences, Fukushima Medical University, Fukushima 960-8516, Japan; nakanowa@fmu.ac.jp (T.N.); satot-pt@fmu.ac.jp (T.S.); y-shiba@fmu.ac.jp (Y.S.); 2 Department of Rehabilitation, Iwaki City Medical Center, Fukushima 973-8555, Japan; 3 Department of Rehabilitation, Takeda General Hospital, Fukushima 965-8585, Japan; 4 Department of Rehabilitation, Jusendo Kaguyama Hospital, Yuasa Hoonkai Foundation, Fukushima 963-8833, Japan; 5 Department of Rehabilitation, Matsumura General Hospital, Iwaki Saiseikai Foundation, Fukushima 970-8516, Japan; 6 Department of Rehabilitation, Ohtanishinouchi Hospital, Ohta General Hospital Foundation, Fukushima 963-8558, Japan; 7 Department of Occupational Therapy, School of Health Sciences, Fukushima Medical University, Fukushima 960-8516, Japan; a-asao@fmu.ac.jp (A.A.); k-natsu@fmu.ac.jp (N.K.); oga-maki@fmu.ac.jp (M.O.); hhayashi@fmu.ac.jp (H.H.); tsone@fmu.ac.jp (T.S.)

**Keywords:** mild cognitive impairment, inertial measurement unit, instrumented Timed Up and Go, smartphone, turning, cognitive screening, community-dwelling older adults

## Abstract

**Highlights:**

**What are the main findings?**

- Turn 1 time during smartphone-based iTUG was significantly associated with screening-defined mild cognitive impairment (screening-defined MCI) after adjustment for age and sex, and the association remained significant after additional adjustment for maximum walking speed and psychological distress.
- A Turn 1 time ≥ 1.35 s was associated with approximately threefold higher odds of screening-defined MCI; however, this ROC-derived cutoff should be considered a preliminary, data-derived threshold from an exploratory analysis and requires independent validation.

**What are the implications of the main findings?**

- Quantitative assessment of turning using smartphone-based IMU sensors may provide additional information on mobility alterations associated with screening-defined MCI.
- Turn 1 time may provide complementary mobility-related information alongside established cognitive screening measures rather than serving as a standalone screening tool.

**Abstract:**

This study used a cognitive screening instrument rather than a clinical diagnosis to define screening-defined mild cognitive impairment (screening-defined MCI) in community-dwelling older adults. Smartphone-based instrumented Timed Up and Go (iTUG) assessment using inertial measurement unit (IMU) sensors enables quantitative assessment of individual movement components. This cross-sectional study investigated the association between Turn 1 time and screening-defined MCI in 318 community-dwelling older adults aged ≥65 years. Cognitive function was assessed using the Japanese version of the Montreal Cognitive Assessment (MoCA-J); 177 participants with scores ≤ 25 were classified as having screening-defined MCI, and 141 with scores ≥ 26 as without screening-defined MCI. Turn 1 time was significantly longer in participants with screening-defined MCI than in those without screening-defined MCI (median [IQR], 1.40 [1.20–1.50] vs. 1.20 [1.10–1.30] s; *p* < 0.001). Receiver operating characteristic analysis yielded an AUC of 0.694 (95% CI, 0.637–0.751), and the preliminary, data-derived cutoff maximizing the Youden index was 1.35 s (sensitivity, 0.525; specificity, 0.780). After adjustment for age and sex, each 0.1 s increase in Turn 1 time was associated with higher odds of screening-defined MCI (OR, 1.322; 95% CI, 1.176–1.487; *p* < 0.001), while a Turn 1 time ≥ 1.35 s was associated with approximately threefold higher odds of screening-defined MCI (OR, 3.108; 95% CI, 1.832–5.271; *p* < 0.001). The association remained significant after additional adjustment for maximum walking speed and psychological distress (OR per 0.1 s increase, 1.286; 95% CI, 1.135–1.457; *p* < 0.001). Smartphone-based iTUG assessment of turning may provide complementary mobility-related information alongside established cognitive screening measures as part of a broader assessment of community-dwelling older adults. The 1.35 s cutoff should be considered a preliminary, data-derived threshold from an exploratory analysis, should not be used for clinical decision-making, and requires validation in an independent sample before any clinical application can be considered.

## 1. Introduction

The global population is aging rapidly, with marked increases in both the number and proportion of older adults [1]. Dementia is a major cause of disability and dependency in later life and places a substantial burden on individuals, families, healthcare systems, and society [2]. Mild cognitive impairment (MCI) is generally regarded as an intermediate state between normal cognitive aging and dementia and is associated with an increased risk of subsequent cognitive decline and progression to dementia [3]. Therefore, early identification of older adults with possible MCI is important for facilitating further cognitive assessment and implementing appropriate preventive strategies.

Cognitive decline may also manifest as changes in mobility and postural control. Walking is not a purely motor task but involves cognitive processes, particularly attention and executive function [4,5]. Longitudinal studies have shown that slowing of gait may precede subsequent cognitive decline [6]. Furthermore, the coexistence of MCI and slow gait [7], as well as concurrent declines in cognition and gait speed [8], have been associated with an increased risk of dementia. Recent studies have also examined relationships between cognitive performance and physiological factors such as pulmonary function, as well as associations between functional mobility and fall-related outcomes in community-dwelling older adults [9,10]. Accordingly, quantitative assessment of mobility may provide additional information for identifying subtle functional changes associated with cognitive decline.

The Timed Up and Go (TUG) test is a simple and widely used clinical assessment of functional mobility in older adults [11]. The conventional TUG measures the total time required to stand up from a chair, walk a specified distance, turn around, return to the chair, and sit down. Although total TUG time is useful for assessing overall mobility and has been commonly applied to evaluate balance and fall risk [11,12], it provides limited information regarding the individual movement components that constitute the task. Previous studies have demonstrated associations between TUG performance and cognitive function in older adults with normal cognition or MCI [13], while analyses of individual TUG subtasks have suggested that specific movement components may provide additional information on cognitive status [14]. A systematic review and meta-analysis further demonstrated progressive deterioration in TUG performance across MCI and Alzheimer’s disease stages, supporting an association between functional mobility and cognitive impairment [15]. However, total completion time alone does not indicate which specific movement components contribute to altered TUG performance.

Recent advances in wearable and mobile sensor technology have enabled more detailed assessment of TUG performance. Instrumented TUG (iTUG) systems using inertial sensors can quantify not only total TUG time but also movement characteristics that cannot be captured by conventional TUG measurements alone [16]. In particular, smartphone-based iTUG systems provide a potentially accessible approach by using smartphone-integrated accelerometers and gyroscopes to objectively quantify movement without requiring specialized laboratory equipment [16,17].

The Hacaro iTUG system divides the TUG into six components—Stand, Go, Turn 1, Come, Turn 2, and Sit—and automatically calculates the duration of each component [16,17]. Among these components, turning may be relevant to mobility changes associated with cognitive decline. Turning is a complex motor task involving deceleration, reorientation of the body, dynamic postural adjustment, and subsequent acceleration in a new direction. Successful turning requires coordination of the limbs and trunk, as well as visual processing, balance, spatial perception, and executive control [18]. Sunderaraman et al. reported that turning duration during the TUG was associated with executive function and processing speed, supporting the cognitive demands of turning [18]. Furthermore, posture and gait control depend on multisensory information and distributed neural networks involving cortical, subcortical, cerebellar, brainstem, and spinal structures [19]. Neuroimaging studies using fNIRS have also demonstrated frontal and parietal cortical activation during straight walking and turning in older adults [20]. Based on these considerations, the present study focused on Turn 1 time as a potentially informative component of mobility performance related to cognitive function.

Although associations between TUG performance, including individual TUG components, and cognitive function have been reported [13,14,15,18], evidence regarding the discriminative ability of smartphone-based turning measures in relation to cognitive impairment remains limited. Moreover, cutoff values specifically for smartphone-based Turn 1 time in relation to cognitive impairment have not been sufficiently investigated.

Therefore, the present study aimed to examine the association between Turn 1 time measured using smartphone-based iTUG and screening-defined mild cognitive impairment (screening-defined MCI) in community-dwelling older adults. We further evaluated the discriminative ability of Turn 1 time for screening-defined MCI and estimated a cutoff value maximizing the Youden index using receiver operating characteristic (ROC) curve analysis. Associations between other iTUG parameters and cognitive function were examined as secondary analyses.

## 2. Materials and Methods

### 2.1. Participants

The study participants were community-dwelling older adults aged ≥65 years residing in three municipalities in Fukushima Prefecture, Japan (Fukushima City, Minamisoma City, and Kagamiishi Town), who were able to walk independently with or without an assistive device. Transportation assistance from family members or others to attend the assessment venue was permitted. Recruitment was conducted through municipal public announcements, including local newsletters. Individuals certified as requiring long-term care under the Japanese long-term care insurance system were excluded. Those unable to complete the physical function assessments required for the present analysis were also excluded.

**Figure 1:**
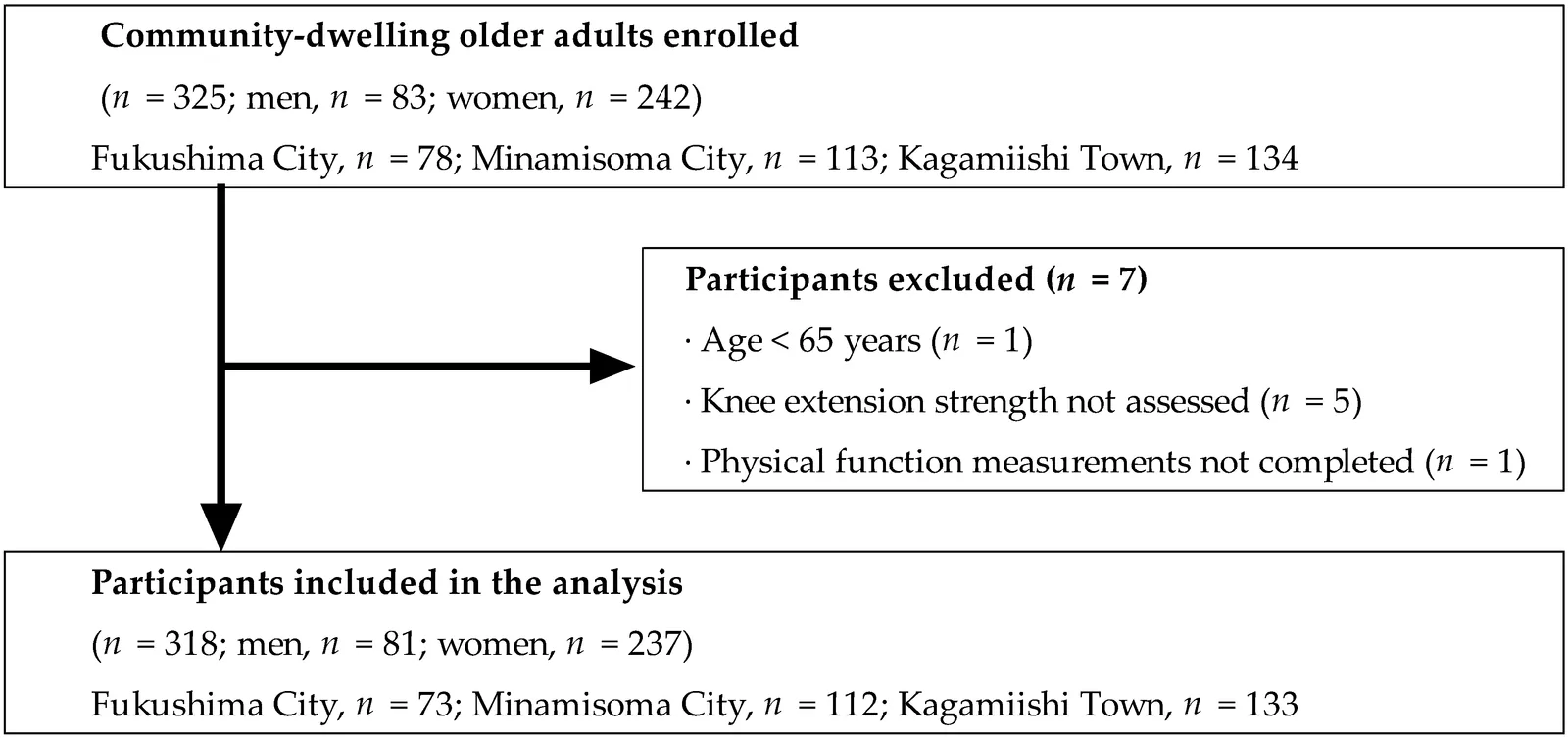
Flowchart of participant selection for the study cohort.

Of the 325 participants initially enrolled, seven were excluded: one was aged <65 years, five did not undergo knee extension strength assessment, and one did not complete the required physical function assessments. Thus, 318 participants (81 men and 237 women) were included in the final analysis. There were no missing data for the variables included in the analyses among these 318 participants. The participant selection process is shown in Figure 1. Descriptive characteristics of participants excluded because of incomplete measurements are provided in Table S3.

### 2.2. Methods

The assessments included demographic and anthropometric characteristics (age, sex, height, weight, and body mass index [BMI]); mobility assessed using the smartphone-based iTUG; physical function (grip strength, knee extension strength, maximum walking speed, and one-leg standing time); cognitive function assessed using the Japanese version of the Montreal Cognitive Assessment (MoCA-J); functional status assessed using the Kihon Checklist (KCL); and psychological distress assessed using the Kessler Psychological Distress Scale (K6). The iTUG assessment included the iTUG score, total iTUG time, individual component times (Stand, Go, Turn 1, Come, Turn 2, and Sit), and three-dimensional trunk acceleration volume (3D-TAV). All assessments were performed by trained examiners using standardized procedures to ensure measurement consistency. For the mobility and physical function assessments, participants wore their usual closed-heel shoes when possible; if these were unavailable, suitable shoes were provided by the investigators.

#### 2.2.1. Demographic and Clinical Characteristics

Age and sex were recorded at the time of assessment. Height and weight were measured with participants wearing light clothing and without shoes. BMI was calculated as body weight in kilograms divided by the square of height in meters (kg/m^2^). Information on falls during the previous year was also collected.

#### 2.2.2. Smartphone-Based iTUG Assessment

Mobility was assessed using the Hacaro iTUG application (Digital Standard Co., Ltd., Osaka, Japan) installed on an iPhone 12 mini (Apple Inc., Cupertino, CA, USA). The smartphone’s built-in inertial sensors, including a three-axis accelerometer and gyroscope, recorded movement during the assessment. The smartphone was placed in a dedicated case attached to a belt and positioned horizontally over the anterior abdomen at the level of the umbilicus, with the screen facing forward.

The TUG was performed by standing up from a 40 cm high chair, walking 3 m to a marker, turning around, walking back to the chair, turning again, and sitting down. Participants were instructed to “Walk as fast as possible without running.” All participants were able to walk independently and performed the iTUG without walking aids. The direction of turning was left to the participant’s discretion and was not systematically recorded or standardized.

The Hacaro iTUG application automatically segmented the TUG into six movement components based on data obtained from the smartphone’s inertial sensors: Stand, Go, Turn 1, Come, Turn 2, and Sit [17]. Angular velocity, orientation, and acceleration were recorded throughout the test. As described previously [17], the segmentation algorithm identifies the movement components using changes in smartphone orientation and angular velocity derived from the gyroscope; pitch angle is used to identify the standing phase, whereas yaw angle and angular velocity are used to identify the walking and turning phases. Turn 1 and Turn 2 represented the turning phases at the 3 m marker and immediately before sitting down, respectively. Thus, Turn 1 time represented the duration of the first turning phase identified automatically at the 3 m marker. The automatically identified movement segments were used without manual correction.

The application provided three categories of outcome measures: (1) temporal measures, including total iTUG time and the duration of each of the six movement components; (2) 3D-TAV; and (3) the iTUG score. Three-dimensional acceleration in the longitudinal, mediolateral, and vertical directions was recorded throughout the iTUG. The 3D-TAV was calculated as the volume of the 95% confidence ellipsoid derived from these three-dimensional acceleration data and was expressed in m^3^/s^6^ [16]. A larger 3D-TAV reflects greater three-dimensional trunk acceleration during the iTUG. The iTUG score is a composite index that incorporates both total iTUG time and 3D-TAV, thereby integrating the temporal and acceleration characteristics of TUG performance; higher scores indicate better mobility performance [16].

Two trials were performed, and the trial with the shorter total iTUG time was selected for analysis; if the total iTUG times were identical, the trial with the higher iTUG score was selected. This selection procedure was used to represent the participant’s best observed mobility performance. Total iTUG time was prioritized because completion time is the primary performance measure of the conventional TUG, whereas the iTUG score was used as a secondary criterion when total times were identical. All iTUG parameters, including the individual component times and 3D-TAV, were obtained from the selected trial. The methodology and component-based analysis of smartphone-based quantitative TUG assessment have been described previously [16,17].

#### 2.2.3. Physical Function Assessments

Grip strength

Grip strength was measured using a digital grip dynamometer (T.K.K.5401, SANKA Co., Ltd., Niigata, Japan). Participants stood with their feet slightly apart and held the dynamometer without allowing it to touch the body. The grip width was adjusted so that the proximal interphalangeal joints were positioned at approximately a right angle during gripping. Two trials were performed using the dominant hand when possible, and the higher value was used for analysis. When the dominant hand could not be assessed because of pain or upper-limb impairment, the non-dominant hand was assessed instead. Grip strength was recorded in kilograms (kg).

Knee extension strength

Knee extension strength was measured using a handheld dynamometer (μTas F-1; Anima Co., Ltd., Tokyo, Japan). Participants sat on a chair with the knee flexed to 90° and their arms crossed in front of the chest. The dynamometer sensor was positioned immediately proximal to the ankle joint of the dominant leg, defined as the leg used to kick a ball. Participants performed a 5 s maximal voluntary isometric knee extension contraction. The maximum force (N) was multiplied by the distance between the knee joint space and the center of the sensor to calculate knee extension torque (N·m). Knee extension strength was then normalized to body mass and expressed as N·m/kg. Two trials were performed when possible, and the higher value was used for analysis. If the dominant leg could not be assessed because of pain or other reasons, the contralateral leg was assessed instead.

Maximum walking speed

Maximum walking speed was assessed over a 14 m walkway consisting of a 10 m measurement section, with a 2 m acceleration zone before and a 2 m deceleration zone after the measurement section. Participants were instructed to walk as fast as possible without running. The time required to walk the central 10 m was measured using a stopwatch from when the leading foot crossed the start line to when it crossed the finish line. Maximum walking speed (m/s) was calculated by dividing 10 m by the recorded walking time. Two trials were performed, and the faster walking speed was used for analysis.

One-leg standing time

One-leg standing time with eyes open was assessed as a measure of static balance. Participants were instructed to stand on their dominant leg, defined as the leg used to kick a ball, for as long as possible. If the dominant leg could not be assessed because of pain or other reasons, the contralateral leg was used. The measurement was terminated when the raised foot touched the floor or the supporting leg, or when external support was required to maintain balance. The maximum measurement time was 60 s.

#### 2.2.4. Cognitive Function Assessment

Cognitive function was assessed using the MoCA-J [21,22]. The MoCA-J is a 30-point cognitive screening instrument that assesses multiple domains, including visuospatial and executive functions, attention, language, memory, and orientation, and can be completed in approximately 10 min. In accordance with the standard MoCA scoring procedure, one point was added to the total score for participants with 12 or fewer years of formal education. Participants with a MoCA-J score ≤ 25 were classified as having screening-defined mild cognitive impairment (screening-defined MCI), whereas those with a score ≥ 26 were classified as not having screening-defined MCI. This cutoff was based on the validation study of the MoCA-J by Fujiwara et al. [22], in which a cutoff of 25/26 demonstrated high sensitivity and specificity for detecting MCI in older Japanese adults. This classification was used for screening purposes and did not represent a clinical diagnosis of MCI.

#### 2.2.5. Kihon Checklist

The KCL is a self-administered questionnaire designed to assess multidimensional health and functional status in older adults [23]. It consists of 25 yes/no items across seven domains: instrumental activities of daily living (five items), physical function (five items), nutritional status (two items), oral function (three items), socialization/housebound status (two items), cognitive function (three items), and depressive mood (five items). Higher total scores indicate poorer health and functional status and a greater risk of frailty.

#### 2.2.6. Psychological Distress Assessment

Psychological distress was assessed using the Kessler Psychological Distress Scale (K6). The K6 is a six-item self-administered questionnaire designed to assess nonspecific psychological distress [24]. Each item is rated on a five-point scale from 0 to 4, yielding a total score ranging from 0 to 24, with higher scores indicating greater psychological distress. The K6 total score was used as a continuous variable in the multivariable logistic regression analysis.

### 2.3. Statistical Analysis

The analyses addressing the primary study objective focused on Turn 1 time. Its association with screening-defined MCI was examined using multivariable binary logistic regression, with Turn 1 time entered as a continuous variable, and its discriminative ability was evaluated using receiver operating characteristic (ROC) curve analysis. To facilitate clinical interpretation, an additional exploratory logistic regression analysis was performed using Turn 1 time dichotomized according to the ROC-derived cutoff value. Because the ROC-derived cutoff was determined and evaluated using the same dataset, no internal or external validation of the cutoff was performed; therefore, the derived threshold should be considered preliminary and exploratory. Secondary analyses included comparisons of participant characteristics, physical function, KCL scores, K6 total scores, and iTUG parameters between participants with and without screening-defined MCI, as well as correlations between MoCA-J scores and iTUG parameters. Age-stratified ROC analyses were also considered exploratory.

The normality of continuous variables was assessed using the Shapiro–Wilk test. Because several variables were not normally distributed, continuous variables are presented as medians with interquartile ranges (IQRs). Comparisons between men and women and between participants with and without screening-defined MCI were performed using the Mann–Whitney *U* test.

Spearman’s rank correlation coefficients (*r*_s_) were calculated to examine the associations between MoCA-J scores and iTUG parameters, including the iTUG score, total iTUG time, individual component times (Stand, Go, Turn 1, Come, Turn 2, and Sit), and 3D-TAV.

The intraday test–retest reliability of Turn 1 time was assessed using measurements obtained from the first and second iTUG trials. The intraclass correlation coefficient (ICC) and corresponding 95% CI were calculated using a two-way mixed-effects model with absolute agreement for single measurements.

Receiver operating characteristic (ROC) curve analysis was performed to evaluate the ability of Turn 1 time to discriminate between participants with and without screening-defined MCI. The area under the curve (AUC) and corresponding 95% confidence interval (CI) were calculated. The cutoff value maximizing the Youden index was estimated, and the corresponding sensitivity and specificity were calculated. In addition to the analysis of the overall sample, exploratory age-stratified ROC analyses were performed for participants aged 65–74 years and those aged ≥75 years.

Multivariable binary logistic regression analyses were performed to examine the association between Turn 1 time and screening-defined MCI. In Model 1, Turn 1 time was analyzed as a continuous variable, with the odds ratio (OR) expressed per 0.1 s increase, after adjustment for age and sex. The linearity of Turn 1 time in the logit was assessed using the Box–Tidwell approach by adding an interaction term between Turn 1 time and its natural logarithm to a logistic regression model adjusted for age and sex. In Model 2, Turn 1 time was dichotomized according to the ROC-derived cutoff value and entered into the model with adjustment for age and sex. Model 2 was considered exploratory because the cutoff value was derived and evaluated using the same dataset. As a sensitivity analysis, Model 3 included Turn 1 time as a continuous variable and was additionally adjusted for maximum walking speed and K6 total score. Age and sex were included as basic demographic covariates because both may be associated with cognitive and mobility performance. Maximum walking speed was additionally included to examine whether the association between Turn 1 time and screening-defined MCI remained after accounting for overall walking performance, while the K6 total score was included to account for the potential influence of psychological distress on cognitive and mobility performance. ORs and corresponding 95% CIs were calculated.

To examine whether the association between Turn 1 time and screening-defined MCI differed by sex, exploratory sex-stratified multivariable logistic regression analyses were additionally performed separately for men and women, with adjustment for age. Turn 1 time was entered as a continuous variable, and the OR was expressed per 0.1 s increase. In addition, a Turn 1 time × sex interaction term was included in a separate model adjusted for age to formally assess whether the association between Turn 1 time and screening-defined MCI differed by sex. Model fit was evaluated using the omnibus test of model coefficients, Nagelkerke R^2^, and the Hosmer–Lemeshow goodness-of-fit test.

To examine whether the association was dependent on the predefined MoCA-J cutoff, an additional sensitivity analysis was performed using the MoCA-J total score as a continuous outcome. Multivariable linear regression analysis was performed with Turn 1 time as the primary independent variable and age and sex as covariates. Turn 1 time was expressed per 0.1 s increase. The unstandardized regression coefficient (B), standardized regression coefficient (β), and corresponding 95% CI were calculated. The assumptions of linear regression were assessed by visual inspection of residual plots, including a histogram and normal P–P plot of standardized residuals and a plot of standardized residuals against standardized predicted values. Multicollinearity was assessed using variance inflation factors.

No formal a priori sample-size calculation was performed because this study was a secondary analysis of data obtained from community-based health assessment events. All eligible participants with complete data required for the analyses were included using a complete-case approach. Because the ROC-derived cutoff was determined and evaluated using the same dataset, no internal or external validation of the cutoff was performed. Therefore, the cutoff value was considered exploratory.

Statistical significance was set at *p* < 0.05. All statistical analyses were performed using IBM SPSS Statistics, version 30 (IBM Corp., Armonk, NY, USA).

### 2.4. Ethics Approval and Consent to Participate

The study protocol was approved by the Research Ethics Committee of Fukushima Medical University (Approval No. General 2022-123). Participation in the community-based health assessment events was voluntary. Before the assessments, participants were provided with a research information sheet and given an oral explanation of the study, including the collection and research use of the assessment data. The study was conducted using an opt-out approach, allowing participants the opportunity to decline the use of their data for research purposes. Accordingly, the requirement for written informed consent was waived by the Research Ethics Committee.

The cognitive assessment booth, where the MoCA-J was administered, was supervised by a psychiatrist at all assessment events. If any concerns regarding a participant’s cognitive or mental status were identified during the assessment process, the psychiatrist provided appropriate clinical guidance as needed. The study was conducted in accordance with the Declaration of Helsinki.

## 3. Results

### 3.1. Participant Characteristics According to Sex

Participant characteristics according to sex are presented in Table 1. The median age of the 318 participants was 75 years (IQR, 72–79 years), and 237 (74.5%) were women. Significant sex differences were observed in height, weight, Stand time, Turn 2 time, grip strength, knee extension strength, MoCA-J scores, and K6 total scores (all *p* < 0.05). No significant sex difference was observed in Turn 1 time (*p* = 0.210).

A descriptive comparison of participants included in the analysis and those excluded because of incomplete measurements is presented in Table S3. Median age was 75 years among the included participants (*n* = 318) and 71.0 years among the excluded participants (*n* = 6), while the corresponding median MoCA-J scores were 25 and 26.5, respectively. Median Turn 1 time was 1.30 s in both groups; however, Turn 1 time was available for only five of the six excluded participants. Given the small number of excluded participants, no formal statistical comparisons were performed.

### 3.2. Comparison of Characteristics Between Participants with and Without Screening-Defined MCI

Characteristics of participants with and without screening-defined MCI are compared in Table 2. Of the 318 participants, 177 were classified as having screening-defined MCI and 141 as not having screening-defined MCI. Participants with screening-defined MCI were significantly older than those without screening-defined MCI (77.0 [74.0–80.5] vs. 74.0 [70.0–76.5] years, respectively; *p* < 0.001), whereas no significant differences were observed in height, weight, or BMI.

Regarding iTUG parameters, participants with screening-defined MCI had a significantly lower iTUG score and a longer total iTUG time than those without screening-defined MCI (both *p* < 0.001). All six component times (Stand, Go, Turn 1, Come, Turn 2, and Sit) were significantly longer in participants with screening-defined MCI (all *p* < 0.05). Turn 1 time was longer in participants with screening-defined MCI than in those without screening-defined MCI (1.40 [1.20–1.50] vs. 1.20 [1.10–1.30] s; *p* < 0.001). Participants with screening-defined MCI also had a significantly lower 3D-TAV than those without screening-defined MCI (*p* < 0.001).

Regarding physical function, knee extension strength, maximum walking speed, and one-leg standing time were significantly lower in participants with screening-defined MCI (all *p* < 0.001), whereas grip strength did not differ significantly between participants with and without screening-defined MCI (*p* = 0.917). The KCL score was significantly higher in participants with screening-defined MCI (*p* = 0.007), whereas the K6 total score did not differ significantly between participants with and without screening-defined MCI (*p* = 0.181).

### 3.3. Correlations Between iTUG Parameters and MoCA-J Scores

Spearman’s rank correlation analysis showed that MoCA-J scores were positively correlated with the iTUG score (*r*_s_ = 0.284, *p* < 0.001) and negatively correlated with total iTUG time (*r*_s_ = −0.380, *p* < 0.001). Among the individual iTUG components, Turn 1 time was negatively correlated with MoCA-J scores (*r*_s_ = −0.336, *p* < 0.001).

### 3.4. Intraday Test–Retest Reliability of Turn 1 Time

The intraday test–retest reliability of Turn 1 time was good, with an ICC of 0.813 (95% CI, 0.772–0.847).

### 3.5. ROC Analysis of Turn 1 Time for Identifying Screening-Defined MCI

**Figure 2:**
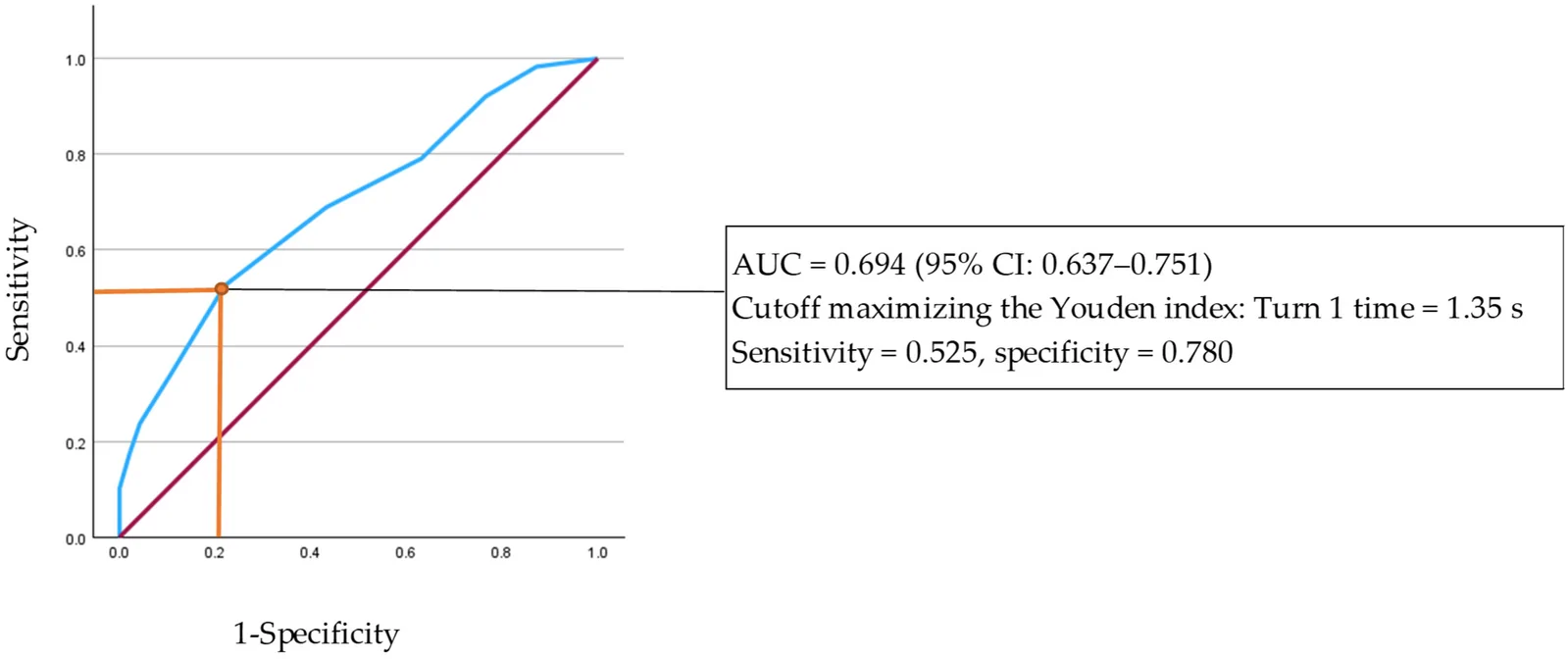
ROC curve of Turn 1 time for identifying screening-defined MCI in community-dwelling older adults. Notes: The blue line represents the ROC curve, and the red line represents the reference line (AUC = 0.5). The orange point indicates the cutoff value that maximized the Youden index.

ROC curve analysis of Turn 1 time for distinguishing between participants with and without screening-defined MCI yielded an AUC of 0.694 (95% CI, 0.637–0.751). The preliminary, data-derived cutoff value maximizing the Youden index was 1.35 s, with a sensitivity of 0.525 and specificity of 0.780 (Figure 2). Because this cutoff was derived and evaluated using the same dataset, it should be interpreted as exploratory and should not be used directly for clinical decision-making. Independent validation in an external cohort is required before any clinical application can be considered.

In exploratory age-stratified analyses, the AUC was 0.630 (95% CI, 0.531–0.730) among participants aged 65–74 years. The cutoff value maximizing the Youden index was 1.05 s (sensitivity, 0.980; specificity, 0.230) (Supplementary Figure S1). Among participants aged ≥75 years, the AUC was 0.702 (95% CI, 0.628–0.777), and the cutoff value maximizing the Youden index was 1.35 s (sensitivity, 0.594; specificity, 0.716) (Supplementary Figure S2).

### 3.6. Association Between Turn 1 Time and Screening-Defined MCI

In Model 1, after adjustment for age and sex, each 0.1 s increase in Turn 1 time was significantly associated with higher odds of screening-defined MCI (OR, 1.322; 95% CI, 1.176–1.487; *p* < 0.001) (Table 3). Age was also positively associated with screening-defined MCI (OR, 1.137; 95% CI, 1.076–1.201; *p* < 0.001), whereas women had lower odds of screening-defined MCI than men (OR, 0.470; 95% CI, 0.257–0.857; *p* = 0.014). The Box–Tidwell analysis showed no evidence of a significant departure from linearity in the association between Turn 1 time and the log-odds of screening-defined MCI (*p* = 0.622).

In exploratory sex-stratified analyses, each 0.1 s increase in Turn 1 time was significantly associated with higher odds of screening-defined MCI after adjustment for age in both men (OR, 1.322; 95% CI, 1.035–1.688; *p* = 0.026) and women (OR, 1.335; 95% CI, 1.166–1.528; *p* < 0.001). The interaction between Turn 1 time and sex was not statistically significant (interaction OR, 1.044; 95% CI, 0.792–1.376; *p* = 0.759), providing no evidence that the association differed by sex.

In an additional exploratory analysis, Turn 1 time was dichotomized using the ROC-derived cutoff value of 1.35 s (Model 2, Table 3). Participants with a Turn 1 time ≥ 1.35 s had approximately threefold higher odds of screening-defined MCI than those with a Turn 1 time < 1.35 s after adjustment for age and sex (OR, 3.108; 95% CI, 1.832–5.271; *p* < 0.001). Age remained positively associated with screening-defined MCI (OR, 1.143; 95% CI, 1.082–1.207; *p* < 0.001), whereas women had lower odds of screening-defined MCI than men (OR, 0.440; 95% CI, 0.242–0.798; *p* = 0.007).

In the sensitivity analysis (Model 3), additional adjustment was made for maximum walking speed and the K6 total score. Turn 1 time remained significantly associated with screening-defined MCI (OR per 0.1 s increase, 1.286; 95% CI, 1.135–1.457; *p* < 0.001). Maximum walking speed (OR, 0.572; 95% CI, 0.222–1.472; *p* = 0.246) and K6 total score (OR, 1.037; 95% CI, 0.946–1.137; *p* = 0.434) were not significantly associated with screening-defined MCI in this model.

To further examine whether the observed association depended on the predefined MoCA-J cutoff, an additional sensitivity analysis was performed using the MoCA-J total score as a continuous outcome (Table S2). In multivariable linear regression analysis adjusted for age and sex, longer Turn 1 time was significantly associated with a lower MoCA-J total score (*B* = −0.299 per 0.1 s increase; 95% CI, −0.429 to −0.169; *p* < 0.001). The model explained 19.9% of the variance in MoCA-J scores (R^2^ = 0.199; adjusted R^2^ = 0.191).

All three logistic regression models were statistically significant based on the omnibus tests (all *p* < 0.001). Nagelkerke R^2^ values were 0.280, 0.251, and 0.287 for Models 1, 2, and 3, respectively. The Hosmer–Lemeshow tests indicated no evidence of poor model fit (all *p* > 0.05).

## 4. Discussion

The present study investigated the association between smartphone-based iTUG parameters and screening-defined MCI in community-dwelling older adults, with particular attention to turning performance. The principal finding was that prolonged Turn 1 time was significantly associated with screening-defined MCI after adjustment for age and sex, and this association remained significant after additional adjustment for maximum walking speed and psychological distress assessed using the K6. These findings suggest that component-based assessment using smartphone-based iTUG, including quantitative assessment of turning, may provide additional information regarding mobility characteristics associated with cognitive function.

Participants with screening-defined MCI showed poorer mobility performance than those without screening-defined MCI. The screening-defined MCI group had a lower iTUG score, longer total iTUG time, and longer times for all six iTUG components (Stand, Go, Turn 1, Come, Turn 2, and Sit). In addition, knee extension strength and maximum walking speed were lower, and one-leg standing time was shorter in the screening-defined MCI group, whereas grip strength did not differ significantly between the groups. These findings are consistent with previous studies demonstrating an association between cognitive decline and deterioration in mobility, particularly gait performance [6,7,8]. Longitudinal studies have shown that slowing of gait may precede subsequent cognitive decline [6]. Furthermore, the coexistence of MCI and slow gait has been associated with an increased risk of developing dementia [7], and concurrent declines in cognition and gait speed have also been linked to a higher risk of dementia [8]. Taken together, these findings support an association between mobility performance and cognitive function and suggest that mobility assessment may provide complementary information regarding cognitive aging.

The conventional TUG is widely used as a simple measure of functional mobility; however, total completion time reflects the combined performance of several distinct motor tasks, including standing up, straight walking, turning, walking back, and sitting down. Consequently, total TUG time alone does not indicate which specific movement components contribute to altered TUG performance. iTUG assessments address this limitation by separating TUG performance into individual components and quantitatively characterizing each component. Previous studies have demonstrated associations between TUG performance and cognitive function in older adults with normal cognition or MCI [13], and specific TUG subtasks have also been associated with distinct cognitive domains [14]. The present study extends these findings by demonstrating that not only total iTUG time but also all six component times differed significantly between participants with and without screening-defined MCI. Moreover, the absolute correlation between MoCA-J score and total iTUG time (*r*_s_ = −0.380) was slightly greater than that for Turn 1 time (*r*_s_ = −0.336), and significant between-group differences were also observed for other components, including Turn 2. Therefore, the present findings do not indicate that Turn 1 is superior to total iTUG time or other iTUG components for identifying screening-defined MCI. Rather, the component-based iTUG assessment enables specific aspects of mobility performance to be examined separately. Among these components, Turn 1 time was of particular interest in the present study because turning requires the integration of multiple motor and cognitive processes. Previous studies have also quantitatively demonstrated associations between TUG performance and cognitive impairment. Mirelman et al. reported that, although total TUG duration did not significantly differ between older adults with and without MCI (8.43 ± 3.72 vs. 7.61 ± 3.78 s, respectively; *p* = 0.121), the duration of the turn-to-walk subtask was significantly longer in those with MCI (2.41 ± 0.67 vs. 2.23 ± 0.61 s; *p* = 0.042) [25]. Poole et al. subsequently demonstrated that quantitative TUG subtask measures were associated with MCI, with walking pace associated with MCI during both usual TUG (OR, 0.71; 95% CI, 0.58–0.86) and dual-task TUG (OR, 0.53; 95% CI, 0.42–0.66) [26]. In a longitudinal study, Ng et al. reported that poorer conventional TUG performance was independently associated with incident MCI or early dementia (adjusted OR, 1.52; 95% CI, 1.01–2.31) and showed an AUC of 0.729 (95% CI, 0.671–0.787) [27]. In the present study, Turn 1 time demonstrated good intraday test–retest reliability (ICC, 0.813; 95% CI, 0.772–0.847). However, because absolute measurement error was not quantified, it remains unclear whether the observed between-group difference of approximately 0.2 s exceeds the measurement error of the method. Therefore, the magnitude of this difference should be interpreted cautiously. Each 0.1 s increase in Turn 1 time was associated with 32.2% higher odds of screening-defined MCI (OR, 1.322; 95% CI, 1.176–1.487), while Turn 1 time showed modest discriminative ability (AUC, 0.694; 95% CI, 0.637–0.751). Although these findings are broadly consistent in demonstrating an association between mobility performance and cognitive impairment, direct comparison of effect sizes should be interpreted cautiously because of differences in study populations, cognitive definitions, TUG protocols, sensor-derived measures, and the scaling of predictor variables across studies.

Consistent with the group comparisons, MoCA-J scores were negatively correlated with Turn 1 time (*r*_s_ = −0.336, *p* < 0.001), indicating that lower cognitive performance was associated with longer turning time. Turning is a complex motor task that requires coordination of the limbs, dynamic postural control, spatial processing, and the integration of sensory information. Sunderaraman et al. reported that turning duration during the TUG was associated with executive function and processing speed, supporting the cognitive demands of turning [18]. Furthermore, gait and postural control depend on distributed neural networks involving cortical, subcortical, cerebellar, and brainstem regions [19], and neuroimaging studies have demonstrated the involvement of frontal and parietal cortical regions during walking in older adults [20]. Taken together, the prolonged Turn 1 time observed in participants with screening-defined MCI may reflect not only reduced physical function and postural control but also greater difficulty in integrating the cognitive and motor processes required for rapid changes in direction. Although the present study cannot identify the underlying neural mechanisms, these findings suggest that turning performance may reflect mobility changes related to cognitive function.

An important finding of the present study was that the association between Turn 1 time and screening-defined MCI remained significant after additional adjustment for selected covariates. In the additionally adjusted model including age, sex, maximum walking speed, and K6 total score, each 0.1 s increase in Turn 1 time was associated with higher odds of screening-defined MCI (OR, 1.286; 95% CI, 1.135–1.457). This finding suggests that the association between Turn 1 time and screening-defined MCI was not fully explained by differences in overall walking performance or psychological distress. The consistency of the findings across the primary and additionally adjusted analyses supports the observed association. The cutoff-based analysis provided complementary exploratory information; however, because the cutoff was derived from the present dataset, it requires validation before clinical application.

ROC analysis yielded an AUC of 0.694 (95% CI, 0.637–0.751) for Turn 1 time. The preliminary, data-derived cutoff maximizing the Youden index was 1.35 s, with a sensitivity of 0.525 and a specificity of 0.780. These findings indicate modest discriminative ability. In particular, the sensitivity of 0.525 indicates that a substantial proportion of participants with screening-defined MCI would not be identified using this threshold. Therefore, Turn 1 time alone would be insufficient as a standalone screening measure for screening-defined MCI. Rather, quantitative turning measures may provide complementary mobility-related information as one component of a broader assessment, alongside established cognitive screening measures and other components of mobility performance. Smartphone-based iTUG has practical advantages in this context because it enables objective and quantitative assessment of specific movement components using a portable device. Thus, Turn 1 time may complement, rather than replace, established cognitive screening instruments.

Exploratory age-stratified ROC analyses among participants aged ≥75 years showed an AUC of 0.702 (95% CI, 0.628–0.777), with a Youden index-derived cutoff of 1.35 s (sensitivity, 0.594; specificity, 0.716), which was identical to that identified in the overall sample. In contrast, the discriminative ability was lower among participants aged 65–74 years (AUC, 0.630; 95% CI, 0.531–0.730), with a Youden index-derived cutoff of 1.05 s (sensitivity, 0.980; specificity, 0.230). These findings suggest that the discriminative performance of Turn 1 time may vary according to age. In particular, the low specificity observed in participants aged 65–74 years suggests limited discriminative utility of this age-specific cutoff in this subgroup. However, because these age-stratified analyses were exploratory and were not externally validated, the findings should be interpreted cautiously. Further studies with larger age-stratified samples and independent validation are needed to determine whether age-specific cutoff values are warranted.

Several limitations should be acknowledged. First, the cross-sectional design precludes conclusions regarding causality or whether prolonged Turn 1 time predicts subsequent cognitive decline or progression to dementia. Longitudinal studies are therefore needed to determine the predictive validity of smartphone-based turning measures.

Second, screening-defined MCI was defined using a single MoCA-J cutoff rather than a comprehensive clinical or neuropsychological diagnosis. Although the 25/26 cutoff was based on a validation study of the MoCA-J in older Japanese adults, some participants may have been misclassified. Nevertheless, the association between longer Turn 1 time and poorer cognitive performance remained significant when the MoCA-J total score was analyzed as a continuous outcome, without relying on the predefined cutoff.

Third, the participants were community-dwelling older adults who were able to walk independently and who performed the iTUG without walking aids, although transportation assistance from family members or others to attend the assessment venue was permitted. The study population may therefore have had relatively preserved mobility, which may limit the generalizability of the findings to frailer older adults or individuals requiring long-term care.

Fourth, although the primary regression models were adjusted for age and sex and the sensitivity analysis additionally accounted for maximum walking speed and psychological distress, comorbidities that may directly affect gait, balance, and turning performance, such as knee osteoarthritis, peripheral neuropathy, and vestibular dysfunction, were not systematically assessed or included in the regression models. Other potentially relevant factors, including physical activity, were also not fully assessed. In addition, information regarding the usual use of walking aids in daily life was not systematically recorded; therefore, its potential influence on Turn 1 performance could not be evaluated. Thus, residual confounding by these unmeasured or incompletely assessed factors cannot be excluded.

Fifth, women comprised a relatively large proportion of the study population, and the smaller number of men may limit the precision of sex-specific estimates. Although exploratory sex-stratified analyses showed similar associations between Turn 1 time and screening-defined MCI in men and women, and the Turn 1 time × sex interaction was not statistically significant, the unequal sex distribution, particularly the smaller number of men, may have limited the statistical power to detect a sex interaction. Therefore, the absence of a statistically significant interaction should not be interpreted as evidence that the association is equivalent between men and women. Further studies with a more balanced sex distribution are needed to examine potential sex differences.

Sixth, turning direction was left to the participant’s discretion and was not systematically recorded or standardized. Therefore, its potential effect on Turn 1 time could not be evaluated in the present study. Future studies should systematically record or standardize turning direction and evaluate its potential influence on turning performance.

Finally, the ROC-derived cutoff of 1.35 s represents a preliminary, data-derived threshold that was determined and evaluated using the same dataset, which may have introduced optimism bias and overestimated its discriminative performance. No internal or external validation was performed; therefore, this threshold should be considered exploratory and requires validation in an independent sample before its clinical usefulness can be established.

## 5. Conclusions

Longer Turn 1 time measured using smartphone-based iTUG was significantly associated with screening-defined MCI in community-dwelling older adults, and this association remained significant after additional adjustment for maximum walking speed and psychological distress. These findings suggest that quantitative assessment of turning using smartphone-based iTUG may provide complementary mobility-related information as one component of a broader assessment. Turn 1 time should not, however, be interpreted as a standalone screening measure for screening-defined MCI. The present study did not establish the superiority of Turn 1 over total iTUG time or other iTUG components. The ROC-derived cutoff of 1.35 s should be considered a preliminary, data-derived threshold from an exploratory analysis and requires validation in an independent sample before its clinical usefulness can be established. Longitudinal studies are also needed to determine the predictive value of individual iTUG components, including Turn 1 time, for subsequent cognitive decline.

## Figures and Tables

**Table 1:** Participant characteristics according to sex.

Variable	Overall (*n* = 318)		Men (*n* = 81)		Women (*n* = 237)		*p* Value
**Demographic characteristics**							
Age (years)	75	(72–79)	76	(72–80)	75	(72–79)	0.330
Height (cm)	153.1	(148.2–159.5)	162.9	(159.5–167.4)	151.0	(146.9–154.6)	<0.001
Weight (kg)	53.8	(47.9–60.5)	62.0	(56.4–68.2)	51.7	(46.5–56.5)	<0.001
BMI (kg/m^2^)	23.0	(20.7–25.0)	23.8	(21.8–25.5)	22.8	(20.4–24.8)	0.070
**iTUG parameters**							
iTUG score	126.3	(102.3–161.4)	134.5	(104.4–168.6)	124.0	(101.2–158.3)	0.173
iTUG time (s)	6.80	(6.30–7.50)	6.90	(6.40–7.60)	6.80	(6.30–7.50)	0.594
Stand time (s)	1.00	(0.80–1.20)	0.90	(0.70–1.20)	1.10	(0.80–1.20)	0.037
Go time (s)	1.40	(1.20–1.60)	1.40	(1.20–1.60)	1.40	(1.20–1.60)	0.988
Turn 1 time (s)	1.30	(1.10–1.40)	1.30	(1.15–1.50)	1.30	(1.10–1.40)	0.210
Come time (s)	1.20	(1.00–1.40)	1.10	(0.90–1.40)	1.20	(1.00–1.40)	0.076
Turn 2 time (s)	0.90	(0.80–1.10)	1.00	(0.90–1.20)	0.90	(0.80–1.10)	0.002
Sit time (s)	1.00	(0.90–1.20)	1.00	(0.90–1.30)	1.00	(0.90–1.20)	0.201
3D-TAV (m^3^/s^6^)	1071.8	(692.1–1683.8)	1199.9	(718.5–1826.7)	1022.1	(671.0–1627.8)	0.131
**Physical function**							
Grip strength (kg)	24.3	(21.4–28.7)	33.6	(30.0–36.7)	22.9	(20.7–24.9)	<0.001
Knee extension strength (N·m/kg)	1.40	(1.17–1.69)	1.60	(1.32–1.83)	1.36	(1.16–1.60)	<0.001
Maximum walking speed (m/s)	1.94	(1.73–2.12)	1.95	(1.76–2.13)	1.93	(1.73–2.12)	0.893
One-leg standing time (s)	28.1	(9.8–60.0)	22.0	(7.1–60.0)	31.2	(10.7–60.0)	0.130
**Cognitive, frailty, and psychological assessments**							
MoCA-J (points)	25	(22–27)	25	(21–26)	25	(22–28)	0.013
KCL score (points)	5	(3–7)	4	(3–6.5)	5	(3–7)	0.114
K6 total score (points)	1	(0–3)	1	(0–2)	1	(0–3)	0.038

Notes: Values are presented as median (interquartile range [IQR]). *p* values were calculated using the Mann–Whitney *U* test. Abbreviations: BMI, body mass index; iTUG, instrumented Timed Up and Go; 3D-TAV, three-dimensional trunk acceleration volume; MoCA-J, the Japanese version of the Montreal Cognitive Assessment; KCL, Kihon Checklist; K6, Kessler Psychological Distress Scale.

**Table 2:** Comparison of characteristics between participants with and without screening-defined MCI.

Variable	Without Screening- Defined MCI (*n* = 141)		With Screening-Defined MCI (*n* = 177)		*p* Value
**Demographic characteristics**					
Age (years)	74.0	(70–76.5)	77.0	(74–80.5)	<0.001
Height (cm)	153.6	(149.3–159.1)	152.4	(147.5–159.9)	0.293
Weight (kg)	54.1	(47.5–60.9)	53.6	(48.5–60.5)	0.980
BMI (kg/m^2^)	22.7	(20.6–25.0)	23.1	(20.9–25.1)	0.427
**iTUG parameters**					
iTUG score	135.1	(109.1–170.2)	116.2	(92.4–152.0)	<0.001
iTUG time (s)	6.50	(6.10–7.00)	7.10	(6.50–7.90)	<0.001
Stand time (s)	0.90	(0.70–1.20)	1.10	(0.80–1.25)	0.019
Go time (s)	1.40	(1.20–1.50)	1.50	(1.20–1.70)	0.010
Turn 1 time (s)	1.20	(1.10–1.30)	1.40	(1.20–1.50)	<0.001
Come time (s)	1.10	(0.90–1.30)	1.20	(1.00–1.40)	0.011
Turn 2 time (s)	0.90	(0.80–1.00)	1.00	(0.90–1.20)	<0.001
Sit time (s)	1.00	(0.90–1.15)	1.10	(0.90–1.25)	0.029
3D-TAV (m^3^/s^6^)	1199.9	(798.6–1834.8)	913.0	(562.6–1516.3)	<0.001
**Physical function**					
Grip strength (kg)	24.3	(21.5–27.6)	24.2	(21.3–29.2)	0.917
Knee extension strength (N·m/kg)	1.48	(1.27–1.79)	1.36	(1.12–1.62)	<0.001
Maximum walking speed (m/s)	2.00	(1.87–2.18)	1.85	(1.65–2.08)	<0.001
One-leg standing time (s)	47.0	(19.8–60.0)	18.4	(7.1–60.0)	<0.001
**Frailty and psychological assessments**					
KCL score (points)	4	(2.5–6)	5	(3–8)	0.007
K6 total score (points)	1	(0–2)	1	(0–4)	0.181

Notes: Values are presented as median (interquartile range [IQR]). *p* values were calculated using the Mann–Whitney *U* test. Abbreviations: MCI, mild cognitive impairment; BMI, body mass index; iTUG, instrumented Timed Up and Go; 3D-TAV, three-dimensional trunk acceleration volume; KCL, Kihon Checklist; K6, Kessler Psychological Distress Scale.

**Table 3:** Logistic regression analysis of the association between Turn 1 time and screening-defined MCI.

Model/Variable	B	SE	OR	95% CI	*p* Value
Model 1: Turn 1 time (continuous model)					
Turn 1 time (per 0.1-s increase)	0.279	0.060	1.322	1.176–1.487	<0.001
Age	0.128	0.028	1.137	1.076–1.201	<0.001
Sex (women vs. men)	−0.756	0.307	0.470	0.257–0.857	0.014
Model 2: Turn 1 time (cutoff-based model)					
Turn 1 time ≥ 1.35 s (vs. <1.35 s)	1.134	0.270	3.108	1.832–5.271	<0.001
Age	0.134	0.028	1.143	1.082–1.207	<0.001
Sex (women vs. men)	−0.822	0.304	0.440	0.242–0.798	0.007
Model 3: Turn 1 time (additionally adjusted model)					
Turn 1 time (per 0.1-s increase)	0.252	0.064	1.286	1.135–1.457	<0.001
Age	0.121	0.029	1.129	1.067–1.194	<0.001
Sex (women vs. men)	−0.798	0.310	0.450	0.245–0.826	0.010
Maximum walking speed (m/s)	−0.559	0.482	0.572	0.222–1.472	0.246
K6 total score (points)	0.037	0.047	1.037	0.946–1.137	0.434
Model fit	Model 1	Model 2	Model 3		
Omnibus test, χ^2^	74.515	65.892	76.560		
*p* value	<0.001	<0.001	<0.001		
Nagelkerke R^2^	0.280	0.251	0.287		
Hosmer–Lemeshow χ^2^	5.497	8.841	6.772		
*p* value	0.703	0.356	0.561		

Notes: Model 1 included Turn 1 time expressed per 0.1 s increase and was adjusted for age and sex. Model 2 included Turn 1 time dichotomized using the ROC-derived cutoff value (≥1.35 s vs. <1.35 s) and was adjusted for age and sex. Model 3 included Turn 1 time expressed per 0.1 s increase and was additionally adjusted for maximum walking speed and K6 total score, in addition to age and sex. Men were used as the reference category for sex. OR, odds ratio; CI, confidence interval; ROC, receiver operating characteristic; K6, Kessler Psychological Distress Scale.

## Data Availability

The datasets generated and/or analyzed during the current study are not publicly available but are available from the corresponding author upon reasonable request.

## Supplementary Materials

The following supporting information can be downloaded at https://www.mdpi.com/article/10.3390/s26185795/s1, Figure S1: ROC curve of Turn 1 time for screening-defined MCI in adults aged 65–74 years; Figure S2: ROC curve of Turn 1 time for screening-defined MCI in adults aged ≥75 years; Table S1: Sex-stratified and interaction analyses of the association between Turn 1 time and screening-defined MCI; Table S2: Sensitivity analysis of the association between Turn 1 time and MoCA-J total score using multivariable linear regression; Table S3: Descriptive comparison of participants included in the analysis and those excluded because of incomplete measurements.
