# Supplementary material for: Association Between Smartphone-Based IMU Turning Performance and Screening-Defined Mild Cognitive Impairment in Older Adults
Source: Sensors (Basel). 2026 Sep 12;26(18):5795. doi: 10.3390/s26185795 (PMC13611128; doi:10.3390/s26185795)
Supplement: Supplementary file 1 [file sensors-26-05795-s001.zip › sensors-4555658-supplementary.pdf]

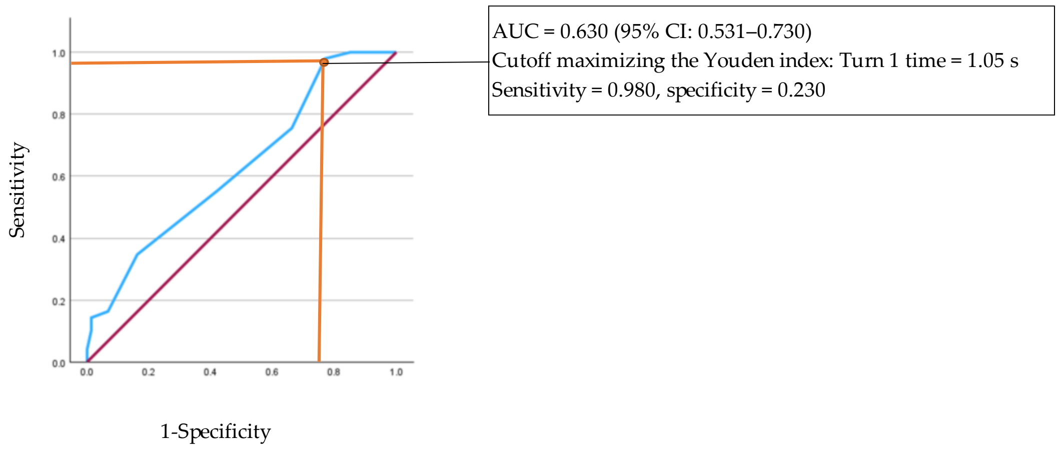

Figure S1. ROC curve of Turn 1 time for screening-defined MCI in adults aged 65–74 years.

Notes: The blue line represents the ROC curve, and the red line represents the reference line (AUC = 0.5). The orange point indicates the cutoff value that maximized the Youden index.

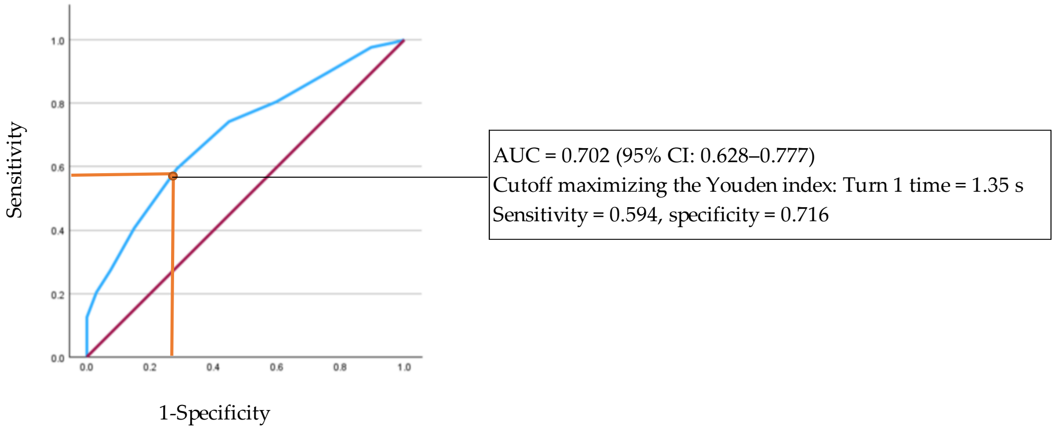

Figure S2. ROC curve of Turn 1 time for screening-defined MCI in adults aged ≥ 75 years.

Notes: The blue line represents the ROC curve, and the red line represents the reference line (AUC = 0.5). The orange point indicates the cutoff value that maximized the Youden index.

Table S1. Sex-stratified and interaction analyses of the association between Turn 1 time and screening-defined MCI

Analysis / Variable	B	SE	OR	95% CI	p value
Sex-stratified analysis: Men (n = 81)					
Turn 1 time (per 0.1-s increase)	0.279	0.125	1.322	1.035–1.688	0.026
Age (per 1-year increase)	0.073	0.053	1.076	0.971–1.193	0.163
Sex-stratified analysis: Women (n = 237)					
Turn 1 time (per 0.1-s increase)	0.289	0.069	1.335	1.166–1.528	< 0.001
Age (per 1-year increase)	0.149	0.034	1.161	1.087–1.240	< 0.001
Sex × Turn 1 interaction analysis (n = 318)					
Turn 1 time (per 0.1-s increase)	0.246	0.123	1.279	1.004–1.629	0.046
Age (per 1-year increase)	0.129	0.028	1.138	1.076–1.202	< 0.001
Sex (women vs. men)	− 1.300	1.801	0.273	0.008–9.300	0.471
Turn 1 time × sex	0.043	0.141	1.044	0.792–1.376	0.759

Note: Sex-stratified multivariable binary logistic regression analyses were adjusted for age. In the interaction analysis, the model included Turn 1 time, age, sex, and the Turn 1 time × sex interaction term. Turn 1 time was expressed per 0.1-s increase. Men were used as the reference category for sex. OR, odds ratio; CI, confidence interval.

Table S2. Sensitivity analysis of the association between Turn 1 time and MoCA-J total score using multivariable linear regression

Variable	B	SE	Standardized β	95% CI for B	*p* value	VIF
Turn 1 time, per 0.1-s increase	−0.299	0.066	−0.241	−0.429 to −0.169	< 0.001	1.113
Age, per 1-year increase	−0.216	0.039	−0.297	−0.292 to −0.140	< 0.001	1.109
Sex, women vs. men	0.485	0.436	0.056	−0.373 to 1.342	0.267	1.010

Model statistics: $R^2 = 0.199$; adjusted $R^2 = 0.191$; $F(3, 314) = 26.009$; $p < 0.001$; $n = 318$.
Note: The MoCA-J total score was entered as a continuous dependent variable. Turn 1 time was expressed per 0.1-s increase. The model was adjusted for age and sex. Men were used as the reference category for sex. B, unstandardized regression coefficient; SE, standard error; β, standardized regression coefficient; CI, confidence interval; VIF, variance inflation factor; MoCA-J, Japanese version of the Montreal Cognitive Assessment.

Table S3. Descriptive comparison of participants included in the analysis and those excluded because of incomplete measurements

Variable	Included (*n* = 318)	Excluded because of incomplete measurements (*n* = 6)
Age, years	75 (72-79)	71.0 (67.5-74.5)
Women, *n* (%)	237 (74.5)	5 (83.3)
MoCA-J score	25 (22-27)	26.5 (26-27)
Turn 1 time, s	1.30 (1.10-1.40)	1.30 (1.10-1.80)[†]

Data are presented as median (interquartile range) or n (%).

†Turn 1 time was available for five of the six participants excluded because of incomplete measurements.
